# Efficacy of xanomeline and trospium chloride in schizophrenia: pooled results from three 5-week, randomized, double-blind, placebo-controlled, EMERGENT trials

**DOI:** 10.1038/s41537-024-00525-6

**Published:** 2024-11-02

**Authors:** Inder Kaul, Sharon Sawchak, Amy Claxton, Colin Sauder, Howard H. Hassman, Rishi Kakar, David P. Walling, Leslie Citrome, Haiyuan Zhu, Andrew C. Miller, Stephen K. Brannan

**Affiliations:** 1 Bristol Myers Squibb, Princeton, NJ USA; 2 CenExel—Hassman Research Institute, Marlton, NJ USA; 3 Segal Trials, Lauderhill, FL USA; 4 CenExel—Collaborative Neuroscience Research, Los Alamitos, CA USA; 5 New York Medical College, Valhalla, NY USA

**Keywords:** Schizophrenia, Schizophrenia

## Abstract

In the 5-week, randomized, double-blind, placebo-controlled EMERGENT-1 (NCT03697252), EMERGENT-2 (NCT04659161), and EMERGENT-3 (NCT04738123) trials, xanomeline and trospium chloride (formerly known as KarXT) significantly improved symptoms of schizophrenia and was generally well tolerated. We pooled data from the EMERGENT trials to further characterize the efficacy of xanomeline/trospium and provide sufficient statistical power to analyze responses in participant subgroups. In pooled analyses, xanomeline/trospium significantly improved Positive and Negative Syndrome Scale (PANSS) total score at week 5 versus placebo (least squares mean difference, –9.9; 95% confidence interval, –12.4, –7.3; *p* < 0.0001; Cohen’s *d* effect size, 0.65). PANSS subscale and Clinical Global Impression–Severity scores also improved significantly with xanomeline/trospium versus placebo. Subgroup analyses consistently favored xanomeline/trospium over placebo regardless of differences in participant age, sex, race, body mass index, and baseline PANSS total score. These results add to existing evidence demonstrating robust and reliable improvements in symptoms with xanomeline/trospium across a broad spectrum of people with schizophrenia.

## Introduction

Muscarinic circuits have been implicated in the pathophysiology of schizophrenia for 60 years and represent a promising alternative to targeting D_2_ dopamine receptors^1^. In trials in Alzheimer’s disease and schizophrenia, treatment with the M_1_/M_4_ preferring muscarinic receptor agonist xanomeline resulted in improvement in symptoms of psychosis without eliciting the motor and metabolic side effects associated with currently available antipsychotics^2,3^; however, other primarily gastrointestinal adverse events precluded further development of xanomeline as a treatment for psychosis.

Xanomeline and trospium chloride combines xanomeline with the peripherally restricted pan muscarinic antagonist trospium chloride with the goal of reducing gastrointestinal side effects^4,5^. The efficacy of xanomeline/trospium (formerly known as KarXT) for the treatment of adults with schizophrenia experiencing acute psychosis was tested in the pivotal EMERGENT-1 (NCT03697252)^6^, EMERGENT-2 (NCT04659161)^7^, and EMERGENT-3 (NCT04738123)^8^ trials. The three 5-week trials were nearly identical, employing a randomized, double-blind, placebo-controlled, flexible-dose design with identical dose levels. In all three trials, the prespecified primary efficacy endpoint of change from baseline to week 5 for xanomeline/trospium compared with placebo in Positive and Negative Syndrome Scale (PANSS) total score was met. Secondary outcome measures, including change from baseline to week 5 for xanomeline/trospium compared with placebo in PANSS positive subscale, negative subscale, Marder negative factor, and Clinical Global Impression-Severity (CGI-S) scores, as well as the proportion of PANSS responders at week 5, also generally favored xanomeline/trospium^6,7,9,10^. In the EMERGENT trials, xanomeline/trospium was well tolerated. The most common side effects were primarily gastrointestinal, related to the activity of xanomeline and trospium at muscarinic receptors (e.g., nausea, vomiting, constipation, dyspepsia), mild to moderate in intensity, and transient in nature^6–8^. Details regarding the pooled safety and tolerability of xanomeline/trospium will be published separately.

Pooling data from individual trials provides enhanced power for analyses based on a larger trial population and affords an opportunity to examine treatment effects in subgroups^11–13^. The 5-week EMERGENT trials are well suited to pooling given the similarity of their trial design and methods. Here, we pooled data from the EMERGENT trials to further characterize the efficacy of xanomeline/trospium in the treatment of schizophrenia.

## Methods

### Participants

The EMERGENT trials enrolled adults (EMERGENT-1, aged 18–60 years; EMERGENT-2 and EMERGENT-3, aged 18–65 years) with a primary diagnosis of schizophrenia based on criteria in the *Diagnostic and Statistical Manual of Mental Disorders*, fifth edition, and confirmed by Mini International Neuropsychiatric Interview for Schizophrenia and Psychotic Disorder Studies (MINI) version 7.0.2. Participants were required to have experienced acute exacerbation of psychotic symptoms necessitating hospitalization within 2 months of screening and be free of all oral antipsychotic medication for at least five half-lives or 1 week, whichever is longer, prior to baseline assessment. Other inclusion criteria included a baseline PANSS total score of 80–120 and a score of ≥4 on at least two positive scale items, as well as a CGI-S score of ≥4. Individuals with a primary diagnosis other than schizophrenia within 12 months prior to screening, a history of treatment resistance to antipsychotic medications, or a reduction in PANSS total score of >20% during the screening period were excluded from the trial^6–8^. A subgroup of participants from across the three trials was identified as exhibiting prominent negative symptoms according to previously published criteria^14^.

### Trial design

The pivotal EMERGENT trials were 5-week, multisite, inpatient, randomized, double-blind, placebo-controlled trials of xanomeline/trospium conducted between September 2018 and December 2022 at 30 sites in the United States and 12 sites in Ukraine (EMERGENT-3 only; Ukraine enrollment ended in February 2022 with the start of the Russia-Ukraine conflict)^6–8^. The pivotal EMERGENT trials were conducted in accordance with the principles of the Declaration of Helsinki, the International Council of Harmonization guidelines for Good Clinical Practice, and the relevant regulations in the countries in which the research was conducted. Protocols and written informed consent were approved by a centralized institutional review board for each trial. The trials followed Consolidated Standards of Reporting Trials (CONSORT) guidelines.

Eligible participants were randomized 1:1 to receive xanomeline/trospium or placebo according to assignments generated by the clinical research organizations Syneos Health (Morrisville, NC, USA; EMERGENT-1) and Veristat (Southborough, MA, USA; EMERGENT-2 and EMERGENT-3). Treatment group assignments were concealed from participants, trial and laboratory personnel, statisticians, and the sponsor. Xanomeline/trospium and placebo were supplied as identical, matching capsules. The flexible dosing schedule began after a 7- to 14-day screening period, beginning with twice-daily 50-mg xanomeline/20-mg trospium and increasing to a maximum of twice-daily 125-mg xanomeline/30-mg trospium by the end of the first week based on tolerability at the investigator’s discretion. Investigators had the option to decrease the dose to 100-mg xanomeline/20-mg trospium twice daily in the event the maximum dose caused unacceptable side effects.

CGI-S and PANSS scores were assessed at baseline and throughout the treatment period. Postbaseline assessments began at week 1 after treatment initiation for CGI-S scores and at week 2 for PANSS scores and continued weekly thereafter, except for the EMERGENT-1 trial where PANSS scores were not assessed at week 3.

### Outcomes

The primary endpoint was change from baseline to week 5 in PANSS total score. The PANSS is a validated, clinician-administered, 30-item scale widely used to assess treatment efficacy in clinical trials of schizophrenia^8,15–17^. The scale comprises 30 items divided into three subscales of positive, negative, and general psychopathology symptoms. Each item is scored on a 7-point scale for a total range of 30–210 points (higher scores indicate greater symptom severity). The prespecified secondary efficacy outcomes were change from baseline to week 5 in PANSS positive subscale, PANSS negative subscale, PANSS Marder negative factor (range, 7–49 for all)^15,18^, and CGI-S score, a measure of overall clinical severity of schizophrenia symptoms (range, 1–7; higher scores reflect more severe symptoms)^19^, and percentage of responders based on CGI-S score (change of 1 or 2 points; EMERGENT-1) and PANSS total score (≥30% reduction from baseline to week 5 in PANSS total score; EMERGENT-2 and EMERGENT-3) criteria.

### Statistical methods

Pooled efficacy analyses were performed in the modified intention-to-treat (mITT) population, which included all enrolled participants who received ≥1 dose of study medication and had a baseline and at least one valid postbaseline PANSS assessment. The primary endpoint was analyzed using a mixed model for repeated measures (MMRM; SAS® software version 9.4 [SAS Institute, Cary, NC, USA]). The model included change from baseline in PANSS total score at week 2, week 3, week 4, and week 5 as the response and treatment group (xanomeline/trospium or placebo), visit (week 2, week 3, week 4, and week 5), interaction between treatment groups and visit, baseline PANSS total score, age, sex, and trial as fixed factors or covariates. Least squares (LS) mean, standard error (SE), and LS mean difference between the xanomeline/trospium and placebo groups at week 5, 95% confidence interval (CI), and the two-sided *p*-value were calculated. A model similar to the MMRM for the primary endpoint was used to analyze change in PANSS positive, PANSS negative, PANSS Marder negative factor, and CGI-S scores. For all analyses, a two-sided *p*-value of less than 0.05 was considered significant. Effect size was assessed using Cohen’s *d* estimate, calculated as the absolute value of the difference in LS mean change in score from baseline to week 5 between xanomeline/trospium and placebo groups divided by the pooled standard deviation (SD) of the change derived from MMRM.

For pooled subgroup analysis, participants were stratified by age based on the trial population median (<45 years or ≥45 years), sex, race (Black or White), ethnicity (Hispanic/Latino or not Hispanic/Latino), country (United States or Ukraine), baseline body mass index (BMI; <30 kg/m^2^ or ≥30 kg/m^2^), and baseline PANSS total score (<95 or ≥95, demarcating the difference between moderate and marked illness; median PANSS total score = 97)^20^. For each subgroup, the primary endpoint of change from baseline in PANSS total score was analyzed using MMRMs similar to the overall population. For subgroup analyses in which the subgroup variable was a term in the model (i.e., age, sex), the term was excluded from the model. Individual MMRM estimates were generated for each subgroup.

Responder analyses were performed using PANSS total score and CGI-S score criteria. The a priori definition of PANSS response was a ≥30% improvement in PANSS total score. Additional response thresholds of ≥20%, ≥40%, and ≥50% were also assessed. Reductions in PANSS total score of 20% to 30% indicate a minimal clinically meaningful change in illness severity, and a score improvement of ≥50% represents much improved^16^. For this analysis, PANSS scores were floor-adjusted by subtracting 30 points from baseline and postbaseline scores. CGI-S responder analysis was performed based on ≥1-, ≥2-, and ≥3-point improvement from baseline to week 5 in CGI-S score. The percentage of PANSS responders and CGI-S responders were compared between xanomeline/trospium and placebo groups using the Wald test, and the 95% CIs of the difference in percentage of responders were estimated using the Newcombe Estimation Method.

## Results

### Patients

**Fig. 1:**
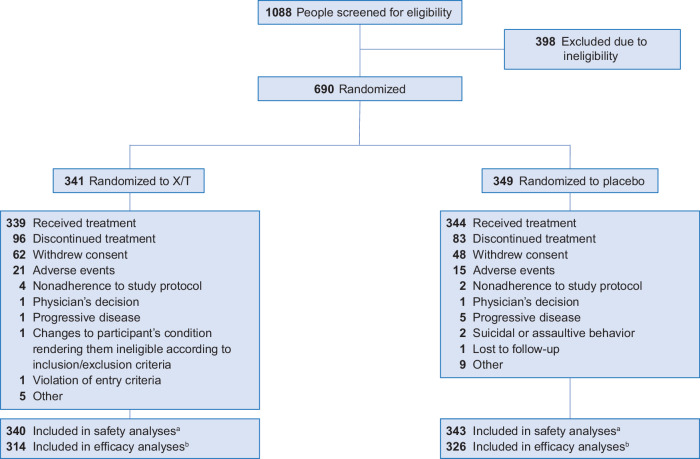
Participant disposition. ^a^The safety population included all participants who received ≥1 dose of trial drug. ^b^The modified intent-to-treat population, used for all efficacy analyses, included all participants randomized who received ≥1 dose of trial drug, had a baseline Positive and Negative Syndrome Scale (PANSS) assessment, and had ≥1 postbaseline PANSS assessment. X/T xanomeline/trospium.

Across the pivotal EMERGENT trials, 1088 people were screened, and 690 were randomized to receive oral xanomeline/trospium or placebo (Fig. 1). The most common reason for discontinuation in both treatment groups was withdrawal of consent, reported in 18.2% and 13.8% of participants in the xanomeline/trospium and placebo groups, respectively. Adverse events were the second most common reason for discontinuation, and rates were relatively low and similar in the xanomeline/trospium and placebo groups (6.2% and 4.3%, respectively). A total of 640 participants (xanomeline/trospium, *n* = 314; placebo, *n* = 326) comprised the mITT population, which was used for the primary efficacy analysis in the individual trials and in pooled efficacy analyses. Baseline demographics and characteristics of the mITT population were generally well balanced between the treatment groups (Table 1). Most participants were male (xanomeline/trospium, 74.2%; placebo, 76.7%) and Black (xanomeline/trospium, 71.7%; placebo, 67.8%) or White (xanomeline/trospium, 26.4%; placebo, 30.1%). The mean (SD) age was 44.6 (10.7) years in the xanomeline/trospium group and 43.7 (11.3) years in the placebo group. Mean (SD) baseline PANSS total scores were 97.5 (9.0) and 97.0 (8.9) in the xanomeline/trospium and placebo groups, respectively. Most participants (xanomeline/trospium, 82.5%; placebo, 96.0%) ended the trial at the highest dose.

**Table 1:** Baseline demographics and characteristics (mITT population).

Parameter	Xanomeline/Trospium (*n* = 314)	Placebo (*n* = 326)
Age (years), mean ± SD	44.6 ± 10.7	43.7 ± 11.3
Sex, *n* (%)		
Male Female	233 (74.2) 81 (25.8)	250 (76.7) 76 (23.3)
Race, *n* (%)		
Asian Black Native Hawaiian or Other Pacific Islander White Other	4 (1.3) 225 (71.7) 1 (0.3) 83 (26.4) 1 (0.3)	2 (0.6) 221 (67.8) 1 (0.3) 98 (30.1) 4 (1.2)
Ethnicity, *n* (%)		
Hispanic or Latino Not Hispanic or Latino Not reported	47 (15.0) 265 (84.4) 2 (0.6)	34 (10.4) 291 (89.3) 1 (0.3)
Country, *n* (%)		
United States Ukraine	295 (93.9) 19 (6.1)	300 (92.0) 26 (8.0)
Weight (kg), mean ± SD	88.9 ± 18.5	87.3 ± 18.6
BMI (kg/m^2^), mean ± SD	29.2 ± 5.5	28.9 ± 5.4
PANSS total score, mean ± SD	97.5 ± 9.0	97.0 ± 8.9
PANSS positive subscale score, mean ± SD	26.6 ± 3.6	26.4 ± 3.4
PANSS negative subscale score, mean ± SD	22.7 ± 3.8	22.6 ± 4.0
PANSS Marder negative factor, mean ± SD	22.4 ± 4.5	22.3 ± 4.6
CGI-S score, mean ± SD	5.1 ± 0.6	5.0 ± 0.6

*BMI* body mass index, *CGI-S* Clinical Global Impression–Severity, *PANSS* Positive and Negative Syndrome Scale.

### Efficacy

**Fig. 2:**
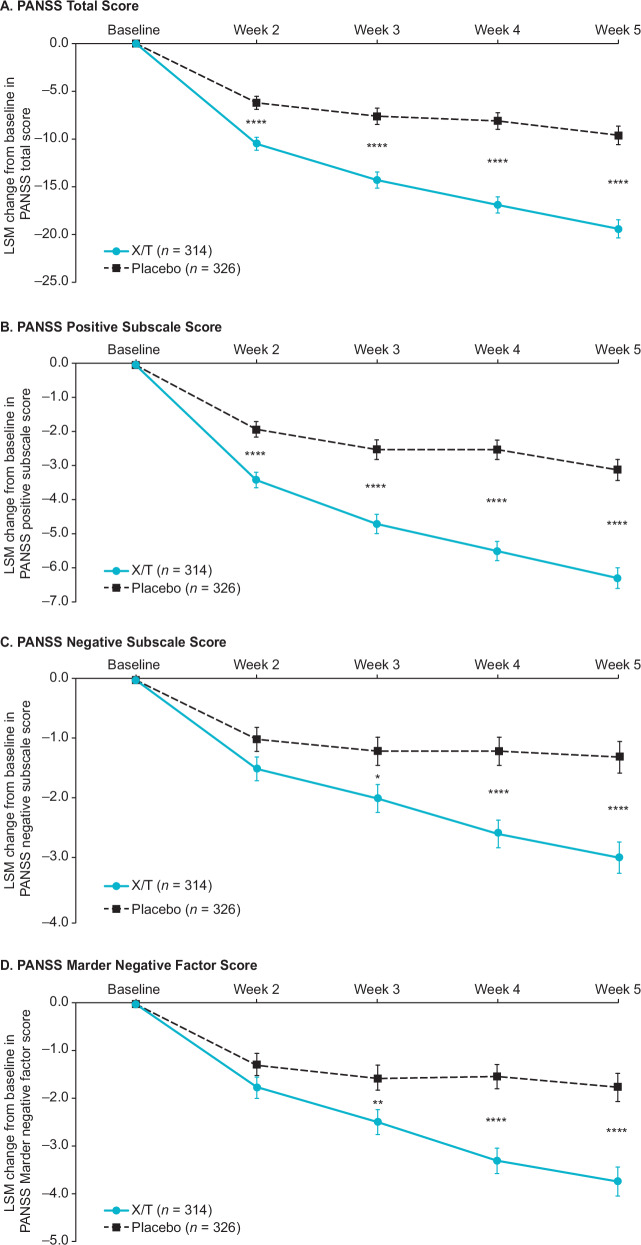
Pooled PANSS scores change from baseline. **A** PANSS total score. **B** PANSS positive subscale score. **C** PANSS negative subscale score. **D** PANSS Marder negative factor score. Values are LSM ± SE. LS mean difference vs. placebo: *p < 0.05; **p < 0.01; ****p < 0.0001. SE standard error, LSM least squares mean, PANSS Positive and Negative Syndrome Scale, X/T xanomeline/trospium.

In the pooled analyses, for the primary endpoint, xanomeline/trospium was associated with a 9.9-point greater reduction from baseline to week 5 in PANSS total score compared with placebo (95% CI –12.4, –7.3; *p* < 0.0001) (Table 2). The PANSS total score Cohen’s *d* effect size of 0.65 demonstrated here is generally representative of results from the individual trials (EMERGENT-1, 0.81; EMERGENT-2, 0.61; EMERGENT-3, 0.60)^7,8,21^. A statistically significant improvement in PANSS total score in the xanomeline/trospium group versus placebo was observed starting at week 2, the earliest timepoint measured, and persisted through week 5 (Fig. 2a).

**Table 2:** Efficacy measures at week 5.

	Xanomeline/Trospium (*n* = 314)	Placebo (*n* = 326)	Difference (95% CI)	Cohen’s *d*	*p* value
Primary endpoint					
PANSS total score	–19.4 (1.0)	–9.6 (1.0)	−9.9 (–12.4, –7.3)	0.65	<0.0001
Secondary outcome measures					
PANSS positive subscale score	–6.3 (0.3)	–3.1 (0.3)	–3.2 (–4.1, –2.4)	0.67	<0.0001
PANSS negative subscale score	–3.0 (0.3)	–1.3 (0.3)	–1.7 (–2.4, –1.0)	0.40	<0.0001
PANSS Marder negative factor score	–3.8 (0.3)	–1.8 (0.3)	–2.0 (–2.8, –1.2)	0.42	<0.0001
CGI-S scale score	–1.1 (0.1)	–0.5 (0.1)	–0.6 (–0.8, –0.4)	0.63	<0.0001
PANSS responders^a^ (≥30% reduction from baseline in PANSS total score)	130/314 (41.4%)	68/326 (20.9%)	20.5 (13.4 to 27.4)	NA	<0.0001

Data are LSM change (SE) from baseline or n/N%.

^a^Floor-adjusted total score (total score minus 30); last observation carried forward. Assessed in participants with available week 5 scores.

*CGI-S* Clinical Global Impression–Severity, *LSM* least squares mean, *SE* standard error, *PANSS* Positive and Negative Syndrome Scale.

**Fig. 3:**
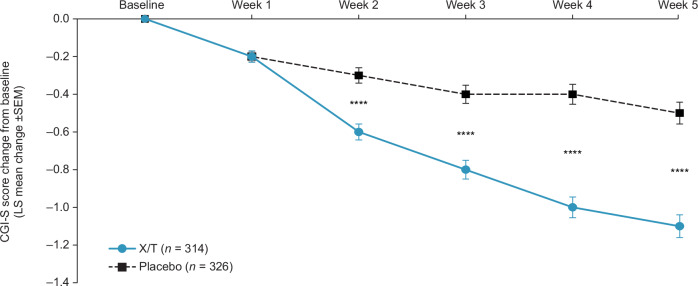
CGI-S score change from baseline. LS mean difference xanomeline/trospium vs. placebo: ****p < 0.0001. CGI-S Clinical Global Improvement‒Severity, LS least squares, PANSS Positive and Negative Syndrome Scale, SEM standard error of the mean, X/T xanomeline/trospium.

Xanomeline/trospium was also associated with larger reductions compared with placebo across all prespecified secondary efficacy outcome measures. At week 5, xanomeline/trospium demonstrated a reduction compared with placebo of −3.2 points in PANSS positive subscale score (95% CI –4.1, –2.4; *p* < 0.0001; Cohen’s *d* effect size, 0.67), −1.7 points in PANSS negative subscale score (95% CI –2.4, –1.0; *p* < 0.0001; Cohen’s *d* effect size, 0.40), −2.0 points in PANSS Marder negative factor score (95% CI –2.8, –1.2; *p* < 0.0001; Cohen’s *d* effect size, 0.42), and −0.6 points in CGI-S score (95% CI –0.8, –0.4; *p* < 0.0001; Cohen’s *d* effect size, 0.63) (Table 2 and Fig. 2b–d and Fig. 3). Statistically significant improvements began at week 2 in the PANSS positive subscale and CGI-S scores and at week 3 in PANSS negative subscale and PANSS Marder negative factor scores; in all measures, significance was maintained from the time of initial observation through week 5.

**Fig. 4:**
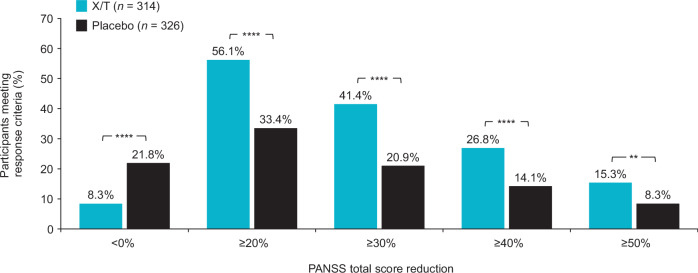
PANSS categorical response rates at week 5. **p < 0.01. ****p < 0.0001. PANSS categorical response rates based on floor-adjusted total score (total score minus 30). PANSS Positive and Negative Syndrome Scale, X/T xanomeline/trospium.

**Fig. 5:**
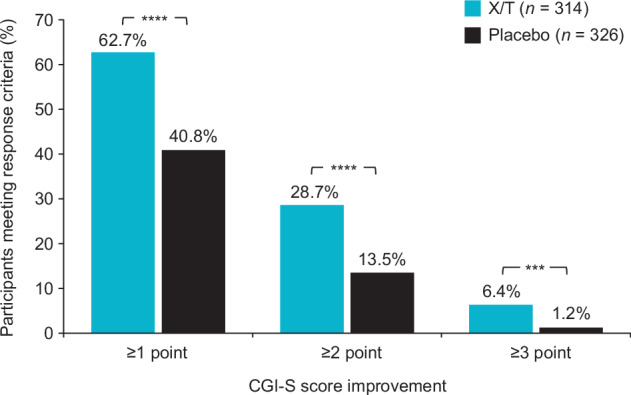
Proportion of participants achieving improvement in CGI-S scores at week 5. ***p < 0.001. ****p < 0.0001. CGI-S Clinical Global Impression–Severity, X/T xanomeline/trospium.

**Fig. 6:**
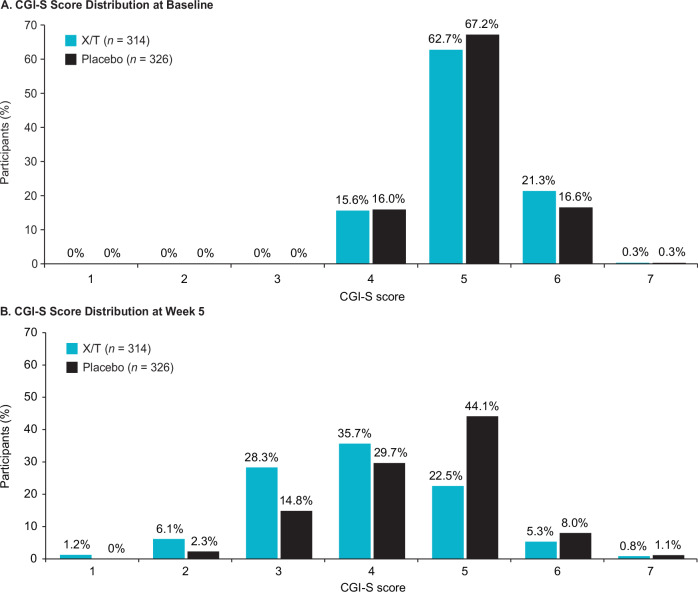
Change in CGI-S score distribution from baseline at week 5^a^. **A** CGI-S score distribution at baseline. **B** CGI-S score distribution at week 5. ^a^CGI-S scores were available at week 5 for 244 participants in the xanomeline/trospium group and 263 participants in the placebo group. CGI-S Clinical Global Impression–Severity, X/T xanomeline/trospium.

Responder analyses based on PANSS total score and CGI-S score criteria favored xanomeline/trospium compared with placebo. The proportion of participants who achieved improvement or worsening from baseline to week 5 in PANSS total score was examined using response criteria thresholds ranging from ≥20% (minimal clinically meaningful change) to ≥50% (much improved)^16^. The percentage of PANSS responders (≥30% improvement) at week 5 was 41.4% in the xanomeline/trospium arm compared with 20.9% in the placebo arm (*p* < 0.0001; Fig. 4); a higher proportion of participants in the xanomeline/trospium arm also met each of the additional PANSS response criteria thresholds than the placebo group. A higher proportion of participants in the xanomeline/trospium group versus the placebo group also had ≥1-, ≥2-, or ≥3-point improvement in CGI-S scale score (Fig. 5). The proportion of responders was approximately one-half higher in the xanomeline/trospium arm compared with placebo at the ≥1-point improvement threshold (62.7% vs. 40.8%), and this difference increased to more than 5-fold at the ≥3-point improvement threshold (6.4% vs. 1.2%) in a small percentage of participants. A similar shift in favor of xanomeline/trospium was observed in CGI-S. At baseline, CGI-S scores for all participants were ≥4, representing ratings of “moderately ill” or greater illness severity^19^. By week 5, 35.7% of people in the xanomeline/trospium group had scores of ≤3 (“mildly ill”, “borderline ill”, or “not at all ill”), compared with 17.1% in the placebo group (Fig. 6).

**Fig. 7:**
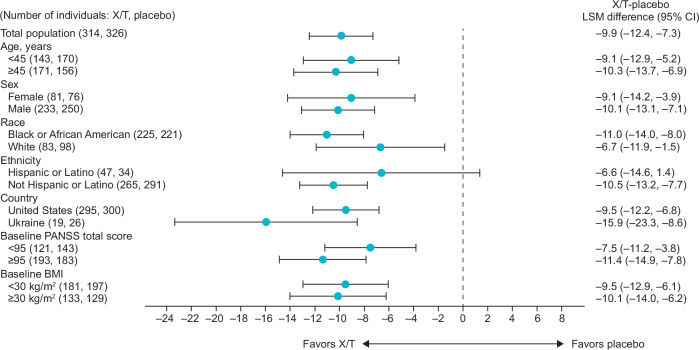
Subgroup analysis of xanomeline/trospium effect on PANSS total score. BMI body mass index, CI confidence interval, LSM least squares mean, PANSS Positive and Negative Syndrome Scale, X/T xanomeline/trospium.

The significant effect of xanomeline/trospium on symptoms of schizophrenia persisted across most subgroups assessed based on demographic and baseline characteristics. As shown in Fig. 7, xanomeline/trospium was associated with a greater reduction from baseline to week 5 in PANSS total score compared with placebo in all subgroups examined, a finding consistent with the significant effects of xanomeline/trospium demonstrated in the three individual trials. Here, a robust xanomeline/trospium effect was minimally impacted by demographic and baseline characteristics, with significant benefits observed in all subgroups based on age, race, gender, BMI, and baseline PANSS score. The one exception was a nonsignificant difference (LS mean difference = ‒6.6; *p* = 0.10) in PANSS total score between xanomeline/trospium and placebo within the Hispanic or Latino subgroup, for which the sample size was small (*n* = 61 at week 5).

## Discussion

In analyses of data pooled from three clinical trials, individuals treated with xanomeline/trospium exhibited significant improvements in symptoms of schizophrenia compared with placebo. These results contribute to existing findings showing reliable, clinically significant reductions in symptoms with xanomeline/trospium across multiple clinical trials and analyses, and suggest the applicability of xanomeline/trospium to the general schizophrenia population regardless of demographic or baseline clinical characteristics^6–8,22–24^. In the pivotal trials, xanomeline/trospium was associated with symptom improvement and was generally well tolerated (reported elsewhere). All three trials met their prespecified primary endpoint: xanomeline/trospium demonstrated significant improvements from baseline to week 5 in PANSS total score of 11.6, 9.6, and 8.4 points over placebo in the EMERGENT-1, EMERGENT-2, and EMERGENT-3 trials, respectively (*p* < 0.0001 for all)^6–8^.

Here, data from the individual trials were pooled in order to examine the effects of xanomeline/trospium in a larger population, an approach that has proven useful in providing additional insight into antipsychotic effects subsequent to the publication of individual trial results^11–13,25,26^. Pooling data provides enhanced statistical power, improves estimation of treatment effects, and allows sufficient power for subgroup analyses. In this post hoc integrated analysis of the three trials, xanomeline/trospium was associated with consistent and significant treatment effect sizes in measures of schizophrenia symptoms and illness severity across the trials, which is not surprising given the results of the individual trials^6–9^. Notably, the PANSS total score effect size of 0.65 observed here parallels results from the pivotal trials in surpassing the median of 0.42 reported in an analysis of 32 antipsychotics^27^.

In addressing whether the observed improvement in negative symptoms was pseudospecific, a post hoc analysis of the pooled EMERGENT trial data showed significant reductions with xanomeline/trospium in negative symptoms among a subset of participants across the three trials with prominent negative symptoms and no predominance of positive symptoms (xanomeline/trospium, *n* = 29; placebo, *n* = 35). Further, the effect of xanomeline/trospium on negative symptoms remained significant compared with placebo after covarying for positive symptoms, depression/anxiety, disorganized thought, or hostility. These results suggest the xanomeline/trospium treatment effect on negative symptoms is promising^28^. However, caution in this interpretation is warranted given that these data are from schizophrenia trials that are of a 5-week duration among individuals with acute exacerbation. Research in larger populations over longer time periods would be necessary to more fully characterize xanomeline/trospium impacts on negative symptoms of schizophrenia.

The present findings align with previous results indicating a benefit for most individuals treated with xanomeline/trospium, as supported by responder analyses based on both PANSS total and CGI-S scores. The two scales provide complementary information regarding treatment benefit: while the PANSS total score reflects symptom severity, a change in CGI-S score provides a measure of clinical significance^15,17^. A ≥2- or ≥3-point change in CGI-S score could represent, for example, an improvement from marked or severe to mild illness, respectively^16^. Such changes were more frequent in the xanomeline/trospium group.

Results of the subgroup analysis indicate a broad benefit from xanomeline/trospium for individuals with varied demographic and baseline clinical characteristics. These results further support two features of xanomeline/trospium treatment observed previously: first, the consistent treatment effect on PANSS total score demonstrated in prior analyses extended to all subgroups examined here. Second, the xanomeline/trospium treatment effects were robust across subgroups, with xanomeline/trospium-associated improvements in PANSS total scores achieving statistical significance compared with placebo in 13 of 14 subgroups examined.

The present analyses have several limitations. First, pooled results reflect the short-term nature of the individual trials; the 52-week EMERGENT-4 and EMERGENT-5 trials will offer insight into long-term efficacy of xanomeline/trospium. Second, the EMERGENT trials lacked an active treatment comparator arm and additional research is needed to directly compare xanomeline/trospium effects against other antipsychotics. Third, although xanomeline/trospium benefits were proportionally greater than those observed in the placebo arm in the subgroup analyses, the absolute numbers of participants in each category were low. Lastly, although the consistent design of the EMERGENT trials mitigated the difficulties inherent in resolving methodological issues among less similar trials^29^, minor differences, such as a change in age criteria from an upper limit of 60 years in EMERGENT-1 to 65 years in the phase 3 trials, may introduce additional variance to the pooled analyses.

In conclusion, in this post hoc pooled analysis of the pivotal EMERGENT trials, xanomeline/trospium was associated with significant and clinically meaningful improvements in symptoms of schizophrenia compared with placebo. These robust treatment effects were observed across a wide range of participant subgroups. The present results are consistent with what was seen in other trials of xanomeline/trospium. Novel treatments with different mechanisms of action and broader efficacy and safety profiles are an area of significant unmet need for people living with schizophrenia. Xanomeline/trospium represents a departure from current antipsychotics in that it has no direct activity at D_2_ dopamine receptors and instead exerts effects through M_1_ and M_4_ muscarinic receptor circuits. The pooled results presented here build on existing support from the individual clinical trials, notably by demonstrating robust treatment effects across a wide range of participant subgroups, for the potential of xanomeline/trospium to be first in a new class of antipsychotic medications based on muscarinic receptor agonism instead of D_2_ dopamine receptor antagonism.

## Data Availability

Data may be requested from the corresponding author, subject to review.
