# Author Correction: Efficacy of xanomeline and trospium chloride in schizophrenia: pooled results from three 5-week, randomized, double-blind, placebo-controlled, EMERGENT trials

**DOI:** 10.1038/s41537-025-00595-0

**Published:** 2025-03-18

**Authors:** Inder Kaul, Sharon Sawchak, Amy Claxton, Colin Sauder, Howard H. Hassman, Rishi Kakar, David P. Walling, Leslie Citrome, Haiyuan Zhu, Andrew C. Miller, Stephen K. Brannan

**Affiliations:** 1 Bristol Myers Squibb, Princeton, NJ USA; 2 CenExel—Hassman Research Institute, Marlton, NJ USA; 3 Segal Trials, Lauderhill, FL USA; 4 CenExel—Collaborative Neuroscience Research, Los Alamitos, CA USA; 5 New York Medical College, Valhalla, NY USA

**Keywords:** Schizophrenia, Schizophrenia

Correction to: *Schizophrenia* 10.1038/s41537-024-00525-6, published online 02 November 2024

In the original version of this article, in Table 2, the difference for the PANSS total score was incorrect and should have been −9.9. In the methods section under “Statistical methods,” the text should have read: “participants who received ≥1 dose of study medication and had a baseline and at…”. Additionally, the text should have read: “...treatment groups and visit, baseline PANSS total score, age, sex, and trial…”. In the results section under “Efficacy,” the PANSS positive subscale data should have been formatted as “PANSS positive subscale score (95% CI –4.1, –2.4; *p* < 0.0001;…)”. This has been corrected in the original article.

